# Does local infiltration analgesia reduce peri-operative inflammation following total hip arthroplasty? A randomized, double-blind study

**DOI:** 10.1186/s12871-017-0354-y

**Published:** 2017-05-03

**Authors:** J. Kuchálik, A. Magnuson, E. Tina, A. Gupta

**Affiliations:** 1 Department of Anesthesiology and Intensive Care, Faculty of Medicine and Health, Örebro University, Örebro, Sweden; 2 Clinical Epidemiology and Biostatistics, School of Medical Sciences, Örebro University, Örebro, Sweden; 3 Clinical Research Laboratory, School of Medical Sciences, Faculty of Medicine and Health, Örebro University, Örebro, Sweden; 4 Perioperative Medicine and Intensive Care, Institution for Physiology and Pharmacology, Karolinska Institutet, and Karolinska University Hospital, Stockholm, Sweden; 5 Department of Anesthesiology and Intensive Care, Karolinska University Hospital, Solna, Stockholm Sweden

**Keywords:** Total hip arthroplasty, Local infiltration analgesia, Postoperative inflammation

## Abstract

**Background:**

Postoperative inflammation following total hip arthroplasty (THA) can lead to delayed mobilization and return of hip function. Our primary aim was to assess whether local infiltration analgesia (LIA) during surgery can prevent postoperative inflammation.

**Methods:**

This is a sub-analysis of data from a broader double-blind study where 56 patients received spinal anaesthesia for THA. Additionally, Group FNB (Femoral Nerve Block) received an ultrasound-guided femoral nerve block using 30 mL of ropivacaine 7.5 mg/mL (225 mg), and 151.5 mL of saline peri-articularly intra-operatively. Group LIA received 30 mL saline in the femoral nerve block and ropivacaine 2 mg/mL, 300 mg (150 mL) + ketorolac 30 mg (1 mL) + adrenaline 0.5 mg (0.5 mL) peri-articularly. After 23 h, the LIA mixture (22 mL) was injected via a catheter placed peri-articularly in Group LIA and 22 mL saline in Group FNB. A battery of pro- and anti-inflammatory cytokines was assessed using a commercially available kit preoperatively and after 4 h and 3 days postoperatively. Additionally, CRP, platelet count and white blood count was determined pre- and postoperatively.

**Results:**

There was a general trend towards an increase in pro-inflammatory cytokines postoperatively, which returned to normal levels after 3 days. IL-6 concentration was significantly lower 4 h postoperatively in Group LIA compared to Group FNB (*p* = 0.015). No other significant differences were found between the groups in other cytokines. CRP levels were significantly higher in Group FNB compared to Group LIA 3 days postoperatively (*p* < 0.001). No other significant differences were seen between the groups.

**Conclusion:**

Local infiltration analgesia has a modest but short-lasting effect on postoperative inflammation in patients undergoing total hip arthroplasty. This is likely to be due to local infiltration of ketorolac and/or local anaesthetics in the LIA mixture. Future studies should be directed towards assessing whether the use of LIA translates into better patient outcomes.

**Trial registration:**

EudraCT Number 2012-003875-20. Registered 3 December 2012

## Background

Postoperative pain following total hip arthroplasty (THA) is commonly managed using different techniques including opioids, local anesthetics, non-steroidal analgesics or regional blocks, alone or in combination. The exact mechanism for postoperative pain following THA is unclear and it is likely that several mechanisms are involved. Trauma causes the migration of inflammatory cells that release cytokines, primarily IL-6, causing a local inflammatory reaction at the site of injury. When cytokines subsequently reach the blood circulation, a systemic reaction may occur which leads to an increase in C-reacting proteins (CRP), serum amyloid A-protein in the liver as well as T- and B-cell activation in the blood and bone marrow [1]. Later, a compensatory anti-inflammatory response causes inhibition of the pro-inflammatory cytokines [2].

In a previous explorative study in patients undergoing total hip arthroplasty, the authors analyzed a battery of 30 cytokines pre- and post-operatively for up to 6 days. They found a significant increase in pro-inflammatory cytokines IL-6, IL-8 and IL-16 in the early postoperative period while IL-12 was reduced [3]. Although several studies have assessed the role of epidural analgesia on postoperative cytokines [4], to our knowledge no studies have examined perioperative inflammation following THA using local infiltration analgesia (LIA). LIA combines ropivacaine, ketorolac and adrenaline in a large volume and is injected systematically, periarticularly, for postoperative pain management. The mechanism of its analgesic effect remains unclear and inhibition of sensory nerves by local anesthetics (LA) as well as reduction in inflammation may be contributing factors. LAs as well as non-steroidal anti-inflammatory drugs (NSAIDs) are known to have anti-inflammatory effects [5], and this may partly explain its analgesic efficacy.

Our hypothesis was that the systematic injection of a mixture of drugs around the hip joint during THA causes a decrease in the inflammatory cytokines, which may be the explanatory mechanism for the analgesic effect of LIA. Thus, the primary aim was to determine plasma concentration of the cytokines IL-6, TNF-α and IL-10 pre- and post-operatively in patients receiving LIA compared to femoral nerve block (FNB). As secondary aims, we analyzed a battery of other pro- and anti-inflammatory cytokines, CRP and thrombocyte and white cell count as an exploratory part of the study in order to determine if other parts of the inflammation cascade are affected by the use of LIA mixture, compared to pain relief using FNB.

## Methods

The study was approved by the Regional Ethical Review Board, Uppsala, Sweden before patient recruitment, and registered in a European Clinical Trials registry (EudraCT Number: 2012-003875-20). All patients gave verbal and written informed consent prior to enrolment. This study is a sub-analysis of data from a study that had the primary aim of assessing pain and recovery following THA (submitted). The present study focuses on the role of loco-regional analgesia on perioperative inflammation. The study was randomized and double blind and all patients were recruited at the Örebro University Hospital during 2013–2015.

Inclusion criteria were: patients 18–80 years age undergoing THA. Patients allergic to local anesthetics, ketorolac or morphine were excluded from the study, as also those on long-term opiate medication prior to study start. No patient was taking non-steroidal anti-inflammatory drugs (NSAIDs) or acetylsalicylic acid prior to surgery, according to hospital routines.

### Preoperative preparation

All patients received paracetamol 1330 mg sustained release and midazolam 0.03 mg/kg orally as premedication, one hour before planned surgery. Cloxacillin 1 g was given orally as prophylactic antibiotic prior to incision.

### Randomization and blinding

Randomization was performed in a 1:1 allocation ratio using computer generated random numbers inserted into opaque, sealed envelopes. The randomization list was kept in a locked cupboard, only to be opened in case of an emergency. The drugs that were to be injected on the following day after surgery were kept in the refrigerator during 24 h.

### Anesthesia and analgesia

In short, an ultrasound probe was used in order to identify the femoral nerve in all patients. Thereafter, and according to group randomization, one of the following solutions was injected.

Group LIA : 30 ml of 0.9% saline in femoral nerve block (FNB) and ropivacaine 0.2% (150 mL), ketorolac 30 mg (1 mL) and adrenaline 5 mg (0.5 mL) systematically, peri-articularly (total 151.5 mL). The technique for LIA has previously been described in details [6].

Group FNB: 30 mL of ropivacaine 7.5 mg/mL was injected in the FNB and 151.5 mL of 0.9% saline systematically, peri-articularly.

Spinal anesthesia with bupivacaine plain 3–3.5 mL (depending on patient’s height) was injected using a 27G spinal needle. Adequacy of spinal block level was established. A multi-hole catheter (Infiltralong 600, 19 G, 600 mm long, Pajunk) was inserted a few cm lateral to the incision, the tip placed intra-articularly and a bacterial filter connected.

### Postoperative pain management

The patients were observed in the recovery ward postoperatively according to hospital routines and thereafter transferred to the general orthopedic ward. All patients received paracetamol sustained release tablets 1330 mg, three times a day. A patient controlled analgesia (PCA) pump was used as rescue medication to administer 1 mg morphine i.v. when needed with a 6 min lock-out time. After 23 h, one of the following study solutions was injected via the intra-articular catheter, according to group randomization.

- Group LIA: 20 ml ropivacaine (7.5 mg/mL), ketorolac 30 mg (1 mL), adrenaline 0.1 mg (1 mL)
- Group FNB: An equal volume (22 mL) of 0.9% saline

The catheter was then removed and sent for culture and sensitivity analysis. After 48 h, the PCA pump was disconnected and all patients then received tramadol 50 mg, maximum four times a day as rescue medication, in addition to paracetamol as above. NSAIDs, aspirin or steroids were not administered perioperatively, except for ketorolac in the LIA mixture, until the study was completed.

### Recordings and measurements

Twenty mL of venous blood was withdrawn for the analysis of cytokines preoperatively, and after 4 h and 3 days postoperatively and for the analysis of CRP, platelet count and total leucocyte count preoperatively and after 3 days. The blood was centrifuged and the plasma was frozen to −70 C for subsequent analysis of cytokines.

### Multiplex cytokine analyses

Plasma concentrations of IFN-γ, IL-6, IL-8, IL-10, IL-12 (p40/p70), IL-1Ra, IL-2R were simultaneous determined using Human Magnetic Custom Luminex® kit (Novex®, Life technologies, Frederick, MD, USA) and for the analytes IL-1β, IL-2, IL-4, IL-5 and TNF-α, a Human high sensitivity MILLIPLEX® MAP kit (EMD Millipore Corporation, Billerica, MA, USA) was used. The assays were performed on undiluted samples in duplicates according to the manufacturer’s protocol. Measurements and analyses were performed using a Luminex 200™ (Luminex Corporation, Austin, TX, USA) and xPONENT® software v 3.1 (Luminex). The standard curve for each cytokine was (pg/mL): IL-10: 19 – 13900, IL-6: 7 – 5000, IL-12: 11 – 8000, IFN-γ: 15 – 10960, IL-1RA: 145 – 106000, IL-2R: 29 – 21250, IL-8: 13 – 9500, IL-1β, IL-2 and IL-5: 0.49 – 2000, IL-4: 1.83 – 7500, TNF-α: 0.43 – 1750.

The primary aim of this study was to determine whether LIA could reduce the inflammatory response to surgery, measured by assay of plasma cytokines IL-6, TNF-α and IL-10 perioperatively, compared to those receiving a femoral nerve block (FNB), which was our standard of care. The secondary aims were to understand whether other plasma cytokines, CRP, thrombocyte and white cell counts might also be affected, positively or negatively, when using LIA compared to FNB.

### Statistics

Continuous variables were summarized with mean and standard deviation (SD) or median and Inter Quartile Range (IQR) when appropriate and categorical variables with percentages. We applied linear mixed model with unstructured correlation structure to evaluate each inflammatory marker with fixed factors: study group, pre-operative measurement of outcome on continuous scale, post-operative time-points on categorical scale and statistical interaction (group x time). If a measured marker were missing because the level was lower than the limit of detection or the sensitivity of the “kit”, the lowest measured value of the marker from the study sample divided by square root of two were used [7]. A second evaluation strategy was used setting these missing values to zero but as no different study conclusions were reached these results are not reported. A maximum of two of 56 patient-samples for a marker measured at any time-point were missing due to reasons other than and not associated with the patient prognosis or treatment. These patients were evaluated in the mixed model analyses under the assumption missing at random. The association measure was mean difference supplemented with 95% confidence interval (CI) between the study groups. The primary outcome measures, cytokines IL-6, TNF-α and IL-10 was corrected for multiple testing with Bonferroni-Holm method. The nine other cytokines were the study secondary outcomes and corrected for multiple testing by the same method. Normality assumptions were evaluated on the mixed models residuals and tested with Shapiro-Wilk test. Log_10_ transformations were used for some markers showing better normality assumption on log scale. These markers are reported with mean ratio, where a mean ratio of 1 implies no mean ratio difference between study groups and a mean ratio of 1.2 means a 20% higher mean in group C compared to LIA. Mann-Whitney test, Chi-2 test or Fischer exact test when appropriate were used to compare study groups for categorical data. *P*-values < 0.05 were considered to be statistically significant. All statistical analyses were done using SPSS version 22.

## Results

**Fig. 1:**
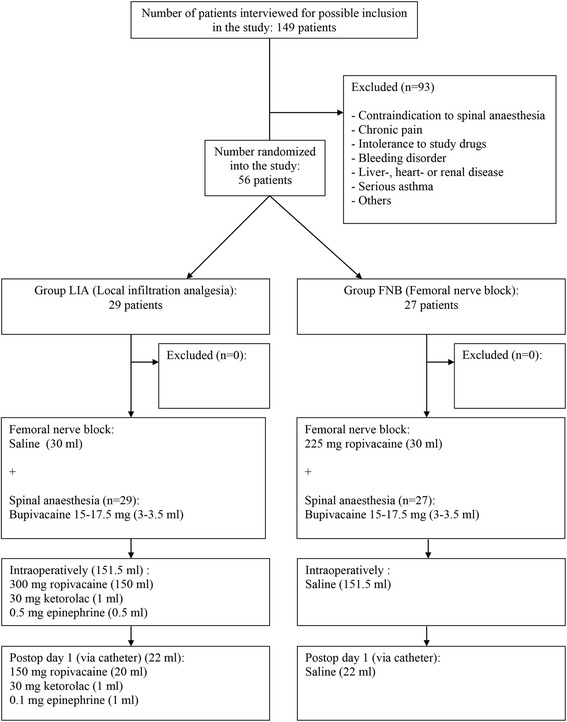
Flow chart for the study. CONSORT diagram on patient recruitment, inclusion and exclusion is shown. LIA = Local infiltration analgesia, FNB = Femoral nerve block

A total of 56 patients were interviewed and agreed to participate in the study. No patient was excluded after randomization and all patients completed the study (Fig. 1). There was no difference seen in the demographic data, operating times, patient characteristics or recovery parameters between the groups (Table 1).

**Table 1:** Demographic data and duration of surgery

	Group LIA (*n* = 29)	Group FNB (*n* = 27)
No of females/males (n)	15/14	15/12
Age, years	64 ± 7	63 ± 8
Weight, kg	84 ± 18	82 ± 19
Height, cm	174 ± 7	173 ± 7
ASA, I/II/III (n)	13/15/1	12/15/0
Operation time, minutes	97 ± 20	92 ± 17

Values are shown as mean ± SD; ASA physical status: I = A normal healthy patient, II = A patient with mild systemic disease, III = A patient with severe systemic disease

*Group LIA* Local infiltration analgesia, *Group FNB* Femoral nerve block

**Fig. 2:**
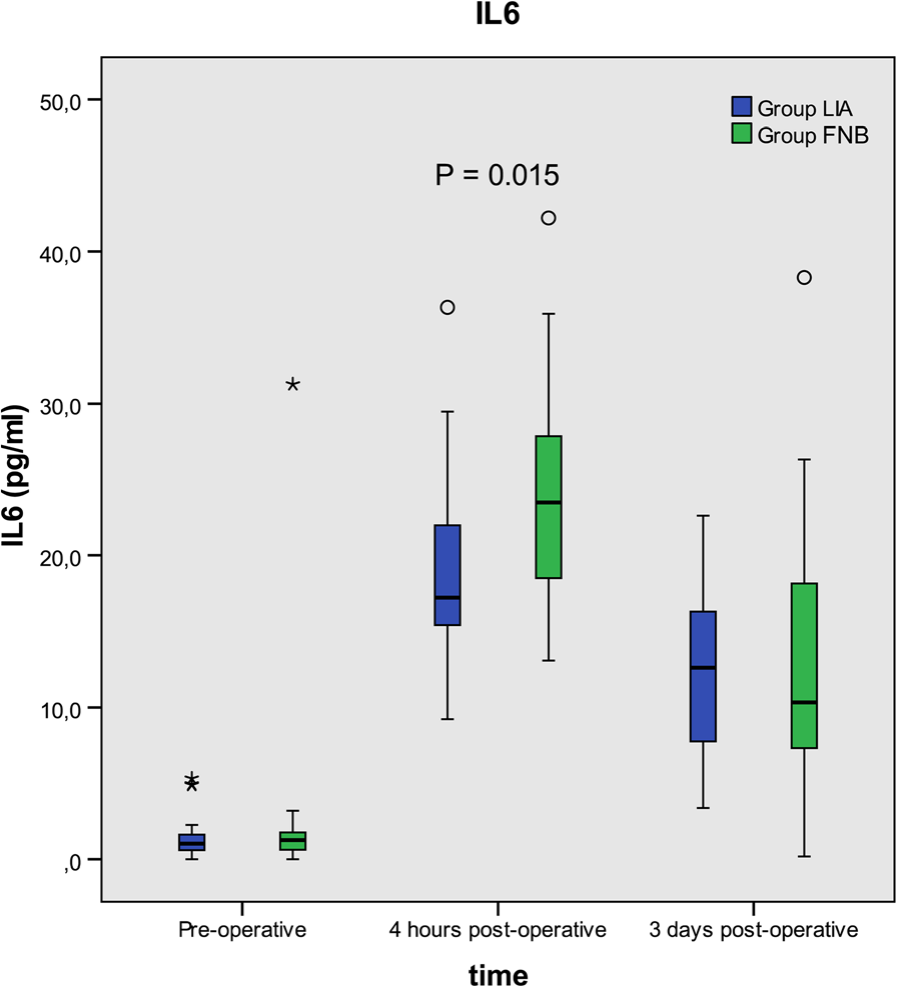
Change in IL-6 over time is shown. LIA = Local infiltration analgesia, FNB = Femoral nerve block

The variation in cytokine concentrations from pre- to post-operative levels are shown in Table 2. The concentration of IL-6 (median, 25th – 75th percentile) was significantly higher in group FNB, 23.5 (18.3–27.9) pg/mL compared to group LIA 17.2 (15.4–23.2) pg/mL at 4 h postoperatively, mean ratio (95% CI) 1.26 (1.08–1.49), *p* = 0.015 (Fig. 2) but not after 3 days postoperatively. No other significant differences were detected between the groups in any other cytokine (IFN-γ, IL-8, IL-10, IL-12 (p40/p70), IL-1Ra, IL-2R, IL-1β, IL-2, IL-4, IL-5 and TNF-α) at 4 or 3 days postoperatively (Table 2, please see separate file).

**Table 2:** Cytokines

	Pre-operative		4 h postoperative				3 days postoperative			
	LIA (N:28)	FNB (N:26)	LIA (N:28)	FNB (N:26)	FNB vs. LIA Mean ratio (95% CI)	*P*-Value	LIA (N:29)	FNB (N:26)	FNB vs. LIA Mean ratio (95% CI)	*P*-Value
IL-6 pg/ml	1.0 (0.6–1.6)	1.3 (0.6–1.8)	17.2 (15.4–23.2)	23.5 (18.3–27.9)	1.26 (1.08–1.49)	0.015	12.6 (7.6–16.4)	10.3 (7.3–18.5)	0.89 (0.59–1.34)	NS
IL-10 pg/ml	3.2 (2.4–3.6)	3.0 (2.5–3.4)	3.7 (3.2–4.4)	3.7 (3.1–4.6)	1.10 (0.95–1.28)	NS	3.4 (2.6–4.0)	3.2 (2.6–3.6)	0.96 (0.90–1.02)	NS
TNF-α pg/ml	5.3 (3.7–6.5)	4.9 (3.3–7.7)	5.4 (2.9–7.3)	4.2 (2.9–5.5)	0.79 (0.61–1.03)	NS	3.6 (2.4–5.8)	3.5 (2.2–8.4)	1.08 (0.74–1.56)	NS
IL-1Rα pg/ml	50.2 (9.7–95.5)	30.2 (6.7–99.9)	96.1 (52.1–282.2)	93.6 (45.3–180.1)	1.01 (0.61–1.66)	NS	96.0 (58.5–134.9)	110.7 (49.0–170.7)	1.19 (0.69–2.06)	NS
IL-1β pg/ml	5.7 (4.1–7.6)	5.6 (3.2–7.4)	3.9 (2.1–6.7)	3.4 (2.6–4.6)	0.95 (0.72–1.26)	NS	3.6 (1.6–5.3)	3.8 (2.2–6.4)	1.36 (0.97–1.92)	NS
IL-2 pg/ml	5.8 (3.2–7.9)	6.4 (2.7–9.4)	4.9 (2.8–8.2)	3.2 (2.1–6.1)	0.89 (0.02–1.73)	NS	3.4 (1.0–6.3)	2.9 (1.6–9.7)	1.31 (0.77–2.22)	NS
IL-2R pg/ml	31.6 (8.8–66.5)	29.7 (20.5–49.2)	42.0 (21.8–61.8)	42.6 (31.2–53.5)	1.03 (0.80–1.32)	NS	46.3 (25.6–85.8)	44.8 (25.7–79.1)	0.90 (0.69–1.16)	NS
IL-4 pg/ml	15.6 (6.9–25.2)	12.2 (4.7–26.3)	10.6 (0.5–17.1)	6.0 (0.5–14.5)	0.77 (0.35–1.67)	NS	0.5 (0.5–15.0)	7.0 (0.5–23.0)	1.37 (0.61–3.07)	NS
IL-5 pg/ml	6.5 (4.3–9.7)	4.6 (3.2–7.1)	5.2 (2.1–7.4)	3.6 (2.5–4.4)	0.88 (0.68–1.15)	NS	4.2 (1.7–6.4)	3.8 (2.6–5.8)	1.30 (0.95–1.78)	NS
IL-8 pg/ml	1.1 (0.5–3.1)	2.2 (0.6–4.3)	6.4 (4.7–10.9)	4.2 (2.3–7.2)	0.30 (0.08–1.16)	NS	2.6 (1.2–3.7)	2.0 (0.8–4.1)	0.86 (0.24–3.09)	NS
IL-12 pg/ml	47.4 (35.4–62.9)	53.6 (37.1–77.6)	46.6 (35.3–64.4)	46.5 (32.5–71.9)	0.91 (0.83–1.00)	NS	50.4 (41.4–69.7)	56.5 (34.4–80.2)	0.89 (0.80–1.00)	NS
IFN-γ pg/ml	0.9 (0.3–1.7)	0.8 (0.2–2.3)	1.6 (0.9–2.4)	1.6 (0.9–3.2)	1.23 (0.93–1.61)	NS	0.9 (0.5–2.0)	0.9 (0.4–2.8)	1.17 (0.89–1.53)	NS

Results of cytokines are shown as median (25th-75th percentile) and mean ratios (95% CI) evaluated on log scale by linear mixed model between study Group C and Group LIA at 4 h and 3 days postoperatively adjusted for pre-operatively measures. P-values for the three study primary outcomes were corrected for multiple comparison with Bonferroni-Holm method. *P*-values for the nine secondary outcomes were corrected with the same method

*NS* non-significant, *CI* confidence interval, *N* number of patients, *Group LIA* local infiltration analgesia, *Group C* Control Group (Femoral nerve block)

**Fig. 3:**
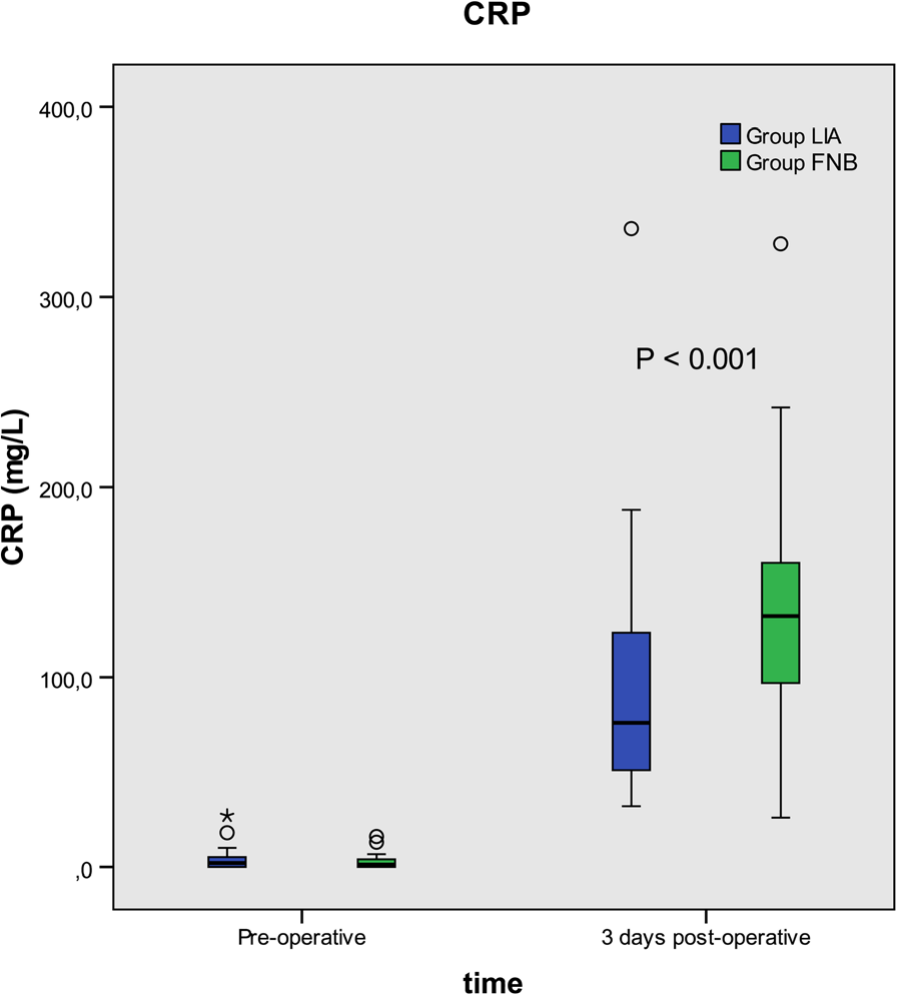
Change in CRP values from pre-operative to postoperative (3 days) is shown. LIA = Local infiltration analgesia, FNB = Femoral nerve block

The total plasma concentration of CRP (median, 25th – 75th percentile) was higher in group FNB, 132 (97–160) mg/L compared to group LIA 76 (50–125) mg/L at 3 days postoperatively, mean ratio (95% CI) 1.60 (1.25–2.06) *p* < 0.001 (Fig. 3). No significant differences were found in total leucocyte or platelet count between the groups (Table 3).

**Table 3:** C-reactive protein (CRP), Platelet count (PC) and Total leucocyte count (TLC)

	Pre-operative		3 days postoperative			
	LIA (N:29)	FNB (N:27)	LIA (N:27)	FNB	FNB vs. LIA Mean ratio (95% CI)	*P*-Value
CRP (mg/L)	2.0 (0.0–5.3)	1.2 (0.0–4.4)	76 (50–125)	132 (97–160) (N:26)	1.60 (1.25–2.06)	<0.001
TLC (10 ^9^/L)	5.8 (4.9–6.8)	6.6 (6.0–7.9)	7.4 (5.9–9.1)	9.2 (7.2–10.4) (N:25)	1.08 (0.97–1.20)	0.17
PC (10 ^9^/L)	244 (214–270)	253 (230–302)	199 (176–221)	227 (203–257) (N:24)	1.07 (0.99–1.16)	0.06

Median (25th -75th percentiles) and mean ratios (95% CI) evaluated on log scale by linear mixed model between study Group FNB and Group LIA at 3 days postoperatively adjusted for pre-operatively measures

*N* number of patients, *Group LIA* local infiltration analgesia, *Group FNB* Femoral nerve block

## Discussion

We found a significantly lower plasma concentration of IL-6 in the LIA group compared to the FNB group at 4 h postoperatively. We also found a lower concentration of CRP at 3 days in the LIA group compared to FNB group. These findings indicate that there is a small but significant effect of LIA in reducing postoperative inflammation following total hip arthroplasty.

Pro- and anti-inflammatory cytokines serve as immune-modulatory molecules that limit potential injury or excess inflammatory reactions during physiologic conditions. Under pathologic conditions, imbalanced cytokines may cause systemic inflammatory responses or immune-suppression due to a shift in the balance between pro- and anti-inflammatory cytokines. This may result in organ dysfunction, immunity and infection, as well as affecting wound healing and pain after surgery. Similarly, musculo-skeletal trauma, as during surgery, causes an inflammatory response that leads, at first, to an elevation of the pro-inflammatory cytokines in the plasma and later, the anti-inflammatory cytokines step in so that a balanced inflammatory response is seen perioperatively. Osteoarthritis of the hip joint results in an increase in pro-inflammatory mediators, specifically IL-6, IL-8 and TNF-α in the synovial fluid [8]. NSAIDs commonly used for the treatment of osteoarthritis decrease IL-6, TNF-alpha and VEGF in the synovial fluid with an improvement in joint pain and function [8]. Thus, it is possible that local infiltration of ketorolac and local anesthetics periarticularly may reduce pain intensity via local anti-inflammatory effects, and could partly explain the known analgesic effect of LIA.

There are several questions that need to be answered from our study. The first is whether the trends in changes in cytokines in the perioperative period during THA are similar to previously published studies. Reikeras et al. studied a battery of 30 cytokines in an exploratory study to better understand the time course of changes in inflammation following THA during spinal anaesthesia [3]. They found a significant increase in IL-6, IL-8 and a decrease in IL-12 at 6 h after THA and return to normal within 6 days. The general trend in our study was similar in both groups even though the absolute values of the cytokines differed between these two studies. The pro-inflammatory cytokines showed an increase post-surgery, and a return towards preoperative values after 3 days. Similarly, the anti-inflammatory cytokines showed only a small change from preoperative values in both groups, even at 3 days.

The next question is whether peripheral or central nerve blocks reduce postoperative inflammation? Previous studies using epidural analgesia (EDA) have shown mixed results with some suggesting a reduction in inflammation while others, not [9, 10]. In several studies from our group comparing epidurals to intravenous morphine analgesia, we could not demonstrate anti-inflammatory effects of the central block as measured by plasma cytokine assay [4, 11]. Similarly, in the present study, we could not demonstrate that FNB reduced pro-inflammatory cytokines; in fact the results were the opposite with LIA technique having a more profound effect in preventing the inflammatory response. While it may be logical to think that a reduction in stress response through better pain management, as when using epidurals or nerve blocks, may reduce inflammation, it is more likely that the degree of musculo-skeletal trauma and not the stress response to surgery per se induces inflammation. Kugisaki et al. found that IL-6 and white blood cell count differed significantly on the first postoperative day in patients having unilateral compared to bilateral knee arthroplasty [12]. Siekmann et al. also showed that open surgery for colo-rectal cancer surgery had a greater impact on cytokine release compared to laparoscopic surgery [11]. Thus, it is likely that more invasive surgery leads to greater degree of inflammation.

The final question is whether LIA, with its component drugs, have any effect on inflammatory mediators during the perioperative phase. To understand this, we compared LIA to FNB and measured a battery of cytokines pre- and post-operatively. We were specifically interested in studying the role of IL-6, TNF-α and IL-10 during surgical trauma. IL-6 has previously been shown to be a sensitive marker of inflammation and increases after both knee and hip arthroplasties, both locally as well as in the plasma [12, 13]. IL-6 is secreted by T-cells and macrophages and stimulates the immune response after trauma or following tissue damage. We found that patients receiving LIA had a significantly lower concentration of plasma IL-6, 4 h postoperatively compared to the FNB group, which persisted after appropriate statistical correction and even logarithmic data transformation. Therefore, the lower IL-6 levels seen in the LIA group, albeit only for a short period of time postoperatively, may reflect a lower degree of inflammation and result from the use of ketorolac or local anaesthetics or both in these patients. Although we injected the LIA mixture even after 24 h, we did not measure cytokines until 2 days later (72 h postoperatively) at which point normality had likely been achieved. Ketorolac and other NSAID’s are potent cyclo-oxygenase inhibitors binding reversibly with this enzyme. If ketorolac is injected intramuscularly/intravenously, it must reach a plasma concentration sufficiently high to promote diffusion of the drug into the site generating the pain. Wirtz et al. showed that there was no difference in cytokine concentration in patients given NSAID’s orally (diclofenac 50 mg × 3) compared to those not receiving NSAIDs [14]. By injecting ketorolac directly into the peri-articular tissues, high concentration of the drug is likely achieved locally and with much lower plasma concentrations. Although some amount of ketorolac is absorbed into the systemic circulation when injected periarticularly and may have systemic anti-inflammatory effects [15], it is more likely that it acts locally since orally administered NSAIDs did not reduce cytokine concentrations [14]. The local concentration of cytokines following THA has been shown to be much higher than systemic concentration [13] and therefore it would have been interesting to measure local cytokine concentrations following LIA, which was not done in the present study. It also remains unclear if there is a dose-response relationship when using ketorolac locally or whether the effect can be prolonged by intermittent injections of ketorolac via a catheter. Further studies should elucidate these important findings.

We did not find any differences in other cytokines (pro- and anti-inflammatory) between the groups and the small but significant difference in IL-6 but not TNF-α may suggest a mild anti-inflammatory effect of the LIA mixture. It is important to remember that several acute phase cytokines such as TNF-α and IL-1(β) have a very short half-life [16] and it is possible that we missed their peak concentration because the first blood sample was taken 4 h postoperatively. We did find a lower CRP concentration in LIA group compared to the FNB group on day 3. CRP is an acute-phase protein of hepatic origin that increases following trauma and inflammation. The release of IL-6 during inflammation also stimulates the production of CRP. Therefore, the lower IL-6 concentration in the LIA group may have lead to a lower CRP, once again supporting our finding that LIA has an anti-inflammatory effect. Hall et al. found a direct correlation between CRP concentration and pain on discharge and a correlation between IL-6 or CRP concentration and the later ability to walk 25 m [17]. This would mean that patients in the LIA group can be mobilized earlier and discharged quicker, which is important to study in the future. Monitoring IL-6 levels may be an important parameter in future studies examining the role of inflammation on postoperative recovery, home discharge and return of bodily functions after surgery.

Amin and Salah compared spinal vs. general anaesthesia and found lower inflammation in the group receiving spinal anaesthesia [18]. In another study, no significant differences in plasma TNF-α or IL-6 (pro-inflammatory cytokines) were found between patients operated under general or regional anesthesia [19]. It is possible that a combination of spinal anaesthesia and locally injected NSAIDs, as in the LIA group in the present study, may provide the correct balance of low pro-inflammatory cytokines in plasma, early mobilization, lower pain intensity and quicker home discharge after surgery.

### Limitations

This is sub-analysis of data from a study that was done to understand the efficacy of LIA on postoperative pain management and therefore we did not perform a formal power analysis to assess the study size. However, several studies published previously have included similar or fewer number of patients (12,13,15). We took blood samples at pre-determined time points partly because the time course of inflammation following THA has previously been characterized (3) and because of financial constraints. Another important limitation of this study is that we did not have a third group of patient that received oral or intravenous NSAIDs. This may have confirmed if the effect of ketorolac and/or local anaesthetics seen by us is via local mechanisms. Finally, it remains unclear whether the reduction in inflammation seen in the present study is the effects of ketorolac or the local anaesthetics injected, both of which are known to have anti-inflammatory effects.

## Conclusion

We found a modest preventive effect of LIA on early postoperative inflammation demonstrated by a lower IL-6 concentration at 4 h as well as a lower CRP concentration 3 days after total hip arthroplasty. This is likely to be a local effect of component drugs, either ketorolac or local anaesthetics that are used in LIA. Further studies should elucidate whether this effect can be translated into improved clinical outcome for our patients.

## Abbreviations

CI: Confidence interval
CRP: C-reacting proteins
FNB: Femoral nerve block
IL-6, 8, 12: Interleukin 6, 8, 12
IQR: Inter-Quartile Range
LIA: Local infiltration analgesia
NSAID: Non-steroidal anti-inflammatory drugs
PNB: Peripheral nerve block
SD: Standard deviation
SPSS: Statistical Package for the Social Sciences
THA: Total hip arthroplasty
TNF-α: Tumor necrosis factor α
